# Nicardipine-Induced Acute Pulmonary Edema: A Rare but Severe Complication of Tocolysis

**DOI:** 10.1155/2014/242703

**Published:** 2014-08-19

**Authors:** Claire Serena, Emmanuelle Begot, Jérôme Cros, Charles Hodler, Anne Laure Fedou, Nathalie Nathan-Denizot, Marc Clavel

**Affiliations:** ^1^Service d'Anesthésie-Réanimation, Hôpital Mère Enfant, 08 avenue Dominique Larrey, 87000 Limoges, France; ^2^Service de Réanimation Polyvalente, CIC 0801, CHU Dupuytren, 02 avenue Martin Luther King, 87000 Limoges, France

## Abstract

We report four cases of acute pulmonary edema that occurred during treatment by intravenous tocolysis using nicardipine in pregnancy patients with no previous heart problems. Clinical severity justified hospitalization in intensive care unit (ICU) each time. Acute dyspnea has begun at an average of 63 hours after initiation of treatment. For all patients, the first diagnosis suspected was pulmonary embolism. The patients' condition improved rapidly with appropriate diuretic treatment and by modifying the tocolysis. The use of intravenous nicardipine is widely used for tocolysis in France even if its prescription does not have a marketing authorization. The pathophysiological mechanisms of this complication remain unclear. The main reported risk factors are spontaneous preterm labor, multiple pregnancy, concomitant obstetrical disease, association with beta-agonists, and fetal lung maturation corticotherapy. A better knowledge of this rare but serious adverse event should improve the management of patients. Nifedipine or atosiban, the efficiency of which tocolysis was also studied, could be an alternative.

## 1. Introduction

In industrialized countries, prematurity is the main cause of perinatal morbidity and mortality and is a major public health issue in obstetrics. Its incidence varies across the country from 5 to 11% [1]. About 75% of premature births are due to spontaneous premature labor [2] which is also the most frequent cause of hospitalization [3]. Tocolytics are used to inhibit uterine contractions so that a fetal lung maturation corticotherapy can be administrated. Whether to prescribe a tocolysis as well as the choice of the molecule to use is still discussed. Massive use of *β*_2_-adrenergic agonists in premature labor treatments induced adverse effects and sometimes severe complications especially acute pulmonary edemas (APE) [4, 5]. Other therapeutic classes are thus more and more preferred. Even if they are prescribed without marketing authorization, calcium channel blockers from the dihydropyridine group, that is, nicardipine (Loxen) and nifedipine (Adalat), have become the first line tocolytic treatments in many French centers [6–9]. After several years of use, rare observations of APE induced by nicardipine are described.

We are reporting four additional observations of APE (from January 2009 to December 2013) occurring in patients hospitalized for premature labor after nicardipine administration. Clinical severity justified hospitalization in intensive care unit (ICU) each time. The objective of this work is to better explain to the physician this rare but classic complication and so to improve its management by preventing inappropriate treatments and especially useless further investigation.

## 2. Case Reports

### 2.1. Case 1

A 31 year-old patient, nullipara, without relevant history and with a single pregnancy, was hospitalized for premature labor at 33 weeks of amenorrhea + 1 day associated with preeclampsia. Her blood pressure had been checked twice a week by a nurse since the 22nd week of amenorrhea as she presented with pregnancy-induced hypertension (PIH). At admission she also presented with a proteinuria higher than 0.3 g/24 h. First she was treated with nicardipine (Loxen) 20 mg × 3/days per os (PO), and 48 hours later as contractions persisted, calcium channel blockers dosage was increased (intravenous (IV) nicardipine, 3.5 mg/h injected with an automatic syringe). Fetal lung maturation corticotherapy was started (two intramuscular (IM) injections of betamethasone 12 mg (Celestene) performed 24 h apart). About 12 hours after tocolytics increase, the patient exhibited a sudden dyspnea with oxygen desaturation at 86% associated with dry cough but without clear signs of acute respiratory distress. She was transferred to ICU for clinical suspicion of pulmonary embolism. Hypoxemia improved under 3 L/min nasal oxygen therapy (SaO_2_ = 96%). Lung examination disclosed crepitant rales at both bases. Arterial blood gas disclosed an uncompensated respiratory alkalosis (pH = 7.50, arterial carbon dioxide tension [PaCO_2_] = 28.2 mmHg, bicarbonates = 21.7 mmol/L, base excess = −1.1 mmol/L, and arterial oxygen tension [PaO_2_] = 107 mmHg) and an increased BNP at 2,474 ng/L. Electrocardiogram (ECG) was normal. A bilateral alveolar-interstitial syndrome was revealed by the chest X-ray. Transthoracic echocardiography (TTE) evidenced a nondilated left ventricle (LV) with a usual systolic function (LV ejection fraction (LVEG) = 70–75%), increased left filling pressures (*E*/*E*′ = 13), and moderately dilated right cavities with pulmonary hypertension (systolic pulmonary artery pressure (SPAP) estimated at 48 mmHg) without paradoxical septum and a noncompliant superior vena cava (SVC) of limited size. Pleural ultrasound showed bilateral pleural effusions measuring around 2 cm. Acute pulmonary edema was diagnosed in light of the X-ray and ultrasound data. Nicardipine was stopped and replaced by atosiban (Tractocile) at a dosage of 8 mL/h IV with an automatic syringe (dilution = 1 ampule, i.e., 37.5 mg in 48 mL).

Following a symptomatic treatment, the disease improved quickly; the 24 h diuresis was equal to 3,050 mL after an IV injection of 80 mg furosemide (Lasilix). Pulmonary disorders disclosed at examination disappeared after 24 h and the chest X-ray was normal again. However the patient still needed oxygen therapy for 36 hours. She was discharged from the ICU at D2 still under furosemide 20 mg/day. At 34 weeks of amenorrhea, that is, D6, emergency cesarean section was decided because of an acute fetal distress with heart rhythm disorders. The newborn was a boy weighing 1,890 grams with an Apgar score of 10/10/10 at 1, 5, and 10 minutes, respectively. No incident occurred after the delivery and the control cardiac and nephrologic assessment performed at 4 months did not disclose any disorder.

### 2.2. Case 2

**Figure 1:**
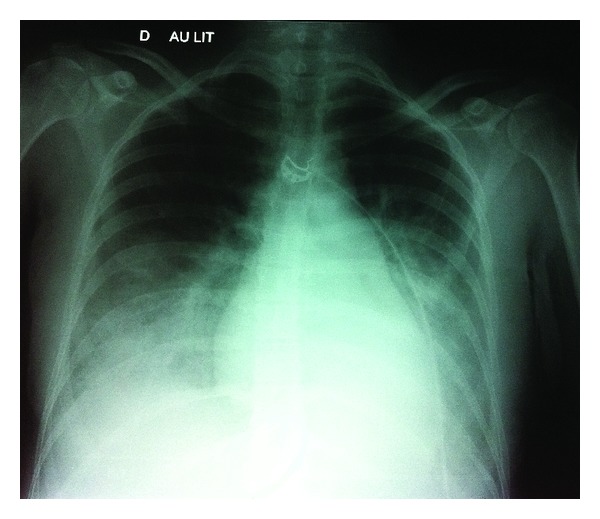
Chest X-ray face-on in prone position (Case 2).

A 28-year-old patient, G3P1, with a spontaneous monochorionic diamniotic twin pregnancy was hospitalized for premature labor at 26 weeks of amenorrhea + 1 day. She successively received a tocolytic treatment with nicardipine (Loxen) IV with an automatic syringe, then atosiban (Tractocile) IV with an automatic syringe, and finally nifedipine (Adalat) PO and salbutamol PO. She also received a fetal lung maturation corticotherapy with betamethasone (Celstene; two IM injections of 12 mg 24 h apart). At D4, because of uterine contraction recurrence, a treatment with nicardipine IV with an automatic syringe at 3 and then 4 mg/h was initiated again. The patient suffered from severe back pain radiating toward the nape, associated with polypnea and desaturation (94% under 15 L/min oxygen in a nonrebreather (NRB) mask). She also presented with photophobia, headaches, and visual disorders induced by nicardipine. Clinical observation revealed no sensory motor deficiency and quick tendon reflexes. Nicardipine therapy was stopped and replaced by atosiban (Tractocile) IV with an automatic syringe. A first TTE disclosed a 70% LVEF, a grade 2 mitral insufficiency, and an aspect similar to an acute cor pulmonale especially a paradoxical septum. In light of the acute respiratory distress with suspected pulmonary embolism, the patient was transferred to ICU. Hemodynamics parameters was stable and there was a jugular turgidity without lower limb edema. The patient still suffered from hypoxia with 90% saturation under 15 L/min oxygen in a NRB mask. A polypnea with supraclavicular indrawing was noted. Examination showed hypoventilation of both bases. Arterial blood gas disclosed an uncompensated respiratory alkalosis (pH = 7.47, PaCO_2_ = 31.2 mmHg, bicarbonates = 22.3 mmol/L, and base excess = −0.9 mmol/L) with hypoxia (PaO_2_ = 59 mmHg under 15 L/min oxygen in a NRB mask) and an increased BNP at 3,686 ng/L. The ECG presented a regular sinus rhythm without conduction or repolarization disorders. Chest X-ray evidenced an alveolar-interstitial infiltrate of both bases (Figure 1). A new TTE confirmed that there was no LV systolic dysfunction but a moderate mitral insufficiency (grade 2); it also revealed increased LV filling pressures (*E*/*E*′ = 15) and pulmonary hypertension (SPAP estimated at 55 mmHg). The aspect of the acute cor pulmonale was not confirmed. Pleural ultrasound revealed two pleural effusions.

We thus assumed that it was an APE induced by the high doses of nicardipine. The patient was treated with diuretics (furosemide Lasilix, 80 mg as bolus then 250 mg/24 h) to which she responded (diuresis of 3,520 mL in 20 h) and with noninvasive ventilation sessions which were not very efficient. As the uterine contractions persisted under atosiban and as a chorioamnionitis was suspected, the patient was intubated and mechanically ventilated to perform an emergency delivery by C-section at 26 weeks of amenorrhea + 5 days. The newborns were two girls weighing 933 and 763 grams hospitalized in neonatal ICU. The patient was extubated 24 hours later as she exhibited respiratory improvement thanks to the diuretics. The patient was discharged at D3 from the ICU still under diuretic treatment. Control echocardiography performed 2 months later was normal.

### 2.3. Case 3

**Figure 2:**
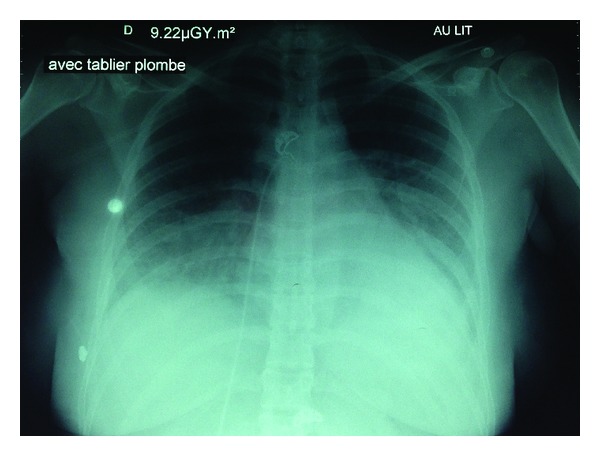
Chest X-ray face-on in prone position (Case 3).

A 28-year-old patient, G2P0, with history of spontaneous miscarriage one year ago (without curettage) was followed in a level 1 maternity for a single pregnancy complicated with an incompetent cervix due to recurrent uterine contractions. She was hospitalized at 29 weeks of amenorrhea + 5 days as uterine contractions were frequent and efficient resulting in cervix dilatation. Premature labor was thus suspected. A tocolytic treatment was started with nicardipine (Loxen) IV with an automatic syringe at doses which rapidly increased up to 4 mg/h. Dyspnea progressively appeared with sensation of thoracic oppression and palpitations. The sensation became more intense during the night of the 5th hospitalization day. The patient presented with febricula at 38.5° and a desaturation at 89% so that nasal oxygen therapy was initiated. At the same time, uterine contractions became more and more intense and fetal lung maturation corticotherapy with betamethasone (Celestene, two IM injections of 12 mg 24 h apart) was started. Laboratory tests revealed an inflammatory syndrome (hyperleukocytosis = 19,000 white blood cells/mm^3^, CRP at 70 mg/L). BNP equaled 660 ng/L with normal troponin. The ECG presented a regular sinus rhythm without any relevant disorder. The TTE disclosed a maintained LVEF with no dilatation of the right cavities. LV filling pressures were normal. SPAP was estimated at 35 + 10 mmHg. As a new desaturation episode occurred, the patient was transferred in ICU. She was still dyspneic with saturation at 89% under oxygen therapy at 6 L/min in a face mask and tended to have high blood pressure. Lung examination disclosed crepitant rales on both lungs especially on the bases. There was no cyanosis or sign of acute respiratory distress. Arterial blood gas revealed an uncompensated respiratory alkalosis (pH = 7.48, PaCO_2_ = 26.7 mmHg, bicarbonates = 19.9 mmol/L, and base excess = −3 mmol/L) associated with hypoxemia (PaO_2_ = 73 mmHg under 6 L/min oxygen). The remaining laboratory tests were normal. Chest X-ray showed bilateral alveolar-interstitial infiltrate in a butterfly distribution without noticeable pneumonia focus (Figure 2). APE induced by tocolysis with nicardipine was diagnosed. The patient was treated with diuretics (furosemide Lasilix, 40 mg PO) and oxygen therapy. Tocolytic treatment was replaced by suppository salbutamol associated with phloroglucinol (Spasfon). The diuretic-induced water and sodium depletion (diuresis of 1,460 mL within 12 h) allowed for a clear improvement of ventilation: polypnea and crepitant rales in the lung disappeared. Under nasal oxygen therapy at 3 L/min, the patient was eupneic with an improved hematosis (PaO_2_ = 187 mmHg). She was discharged from the ICU at D2 still under furosemide 20 mg/day. She gave birth at 35 weeks of amenorrhea + 7 days by vaginal delivery and underwent at 4 months a control TTE that was normal.

### 2.4. Case 4

**Figure 3:**
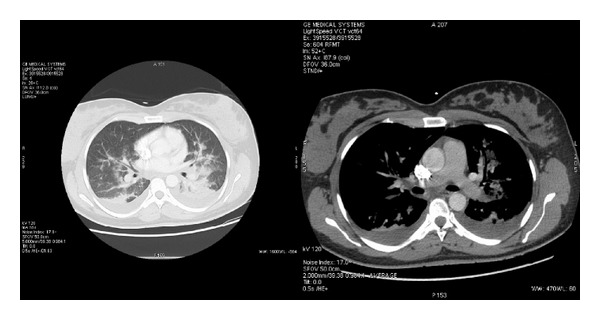
Thoracic angioscan (Case 4).

A 35-year-old patient, primipara, presented with a single pregnancy after* in vitro* fertilization due to altered tubes. She had a history of smoking, which was weaned, substituted hypothyroidism, and a previous* in vitro* fertilization which resulted in an ectopic pregnancy. She was hospitalized in a level 1 maternity for premature labor occurring at 34 weeks of amenorrhea. She received nicardipine (Loxen) which she tolerated well (IV with an automatic syringe at 4 mg/h), associated with suppository salbutamol. Betamethasone was administered (Celestene, two IM injections of 12 mg 24 h apart) for fetal lung maturation. Forty-eight hours after nicardipine administration, the patient suddenly exhibited dyspnea associated with a sensation of thoracic oppression and dry cough with apyrexia. Arterial blood gas disclosed a “shunt” effect (PaO_2_ = 69 mmHg and PaCO_2_ = 27 mmHg). As pulmonary embolism was suspected, a treatment with heparin IV with an automatic syringe, preceded by a bolus, was initiated. The patient was then transferred to the emergency department without relevant complication. Under nicardipine, patient hemodynamics was stable so the treatment was stopped. The patient was apyretic. Regarding ventilation, the patient was eupneic under high flow oxygen therapy (saturation at 100% under 15 L/min in a NRB mask). Arterial blood gas disclosed a compensated metabolic acidosis (pH = 7.42, PaCO_2_ = 29.7 mmHg, bicarbonates = 19.3 mmol/L, and base excess = −4.3 mmol/L) with an impaired hematosis (PaO_2_ at 107 mmHg under 15 L/min oxygen). Anemia at 10.3 g/dL and hyperlactatemia at 3.16 mmol/L were also noticed. The ECG presented a regular sinus rhythm without any relevant disorder. Chest X-ray evidenced bilateral diffuse infiltrative lesions. TTE showed normal size of the LV with a LVEF estimated at 70%. The presence of a minimal mitral insufficiency was noticed and LV filling pressures were normal. SPAP was estimated at 19 + 10 mmHg. Pulmonary embolism diagnosis was ruled out thanks to a thoracic angioscan. It however disclosed a slight bilateral pleural effusion associated with large nonsystematized bilateral alveolar opacities (Figure 3). In light of the tests, APE induced by nicardipine was diagnosed and the patient was transferred to the ICU. Thanks to a symptomatic treatment (oxygen therapy at 6 L/min in face mask) and diuretics (iterative bolus of furosemide for a total dose of 180 mg), the health of the patient rapidly improved. The 24 h diuresis equaled 4,210 mL which allowed for the water and sodium balance to become negative again (−2,928 mL). At discharge, patient was eupneic in ambient air but discrete crepitant rales persisted at both lung bases. She was discharged from the ICU at D2 still under furosemide 40 mg/day. After her delivery, the cardiologic assessment was strictly normal.

Patients' characteristics are reported in Table 1.

## 3. Discussion

**Figure 4:**
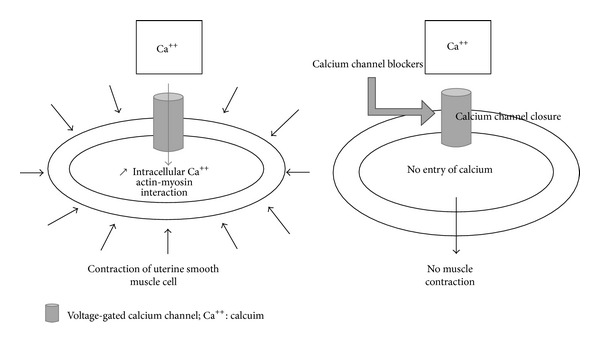
Mode of action of calcium channel blockers.

Premature labour diagnosis was defined in association with uterine contractions (regular and effective) and cervix changes occurring at 22 weeks of amenorrhea and 36 weeks of amenorrhea + 6 D. Without any medical intervention, they lead to premature delivery. All patients had undergone a vaginal palpation and a systematic cervical ultrasound. A 30-minute fetal monitoring was performed on admission and then once a day. Obstetric ultrasound was also performed. The occurrence of uterine contractions which are essential for the labor to begin is regulated by the increase of intracellular calcium concentration in myometrial cells. Calcium channel blockers (CCBs) from dihydropyridine family, that is, nicardipine (Loxen) and nifedipine (Adalat), fix themselves on *α*_1*c*_ subunit of the L-type voltage-gated calcium channels. This way, they block the opening of the channel and prevent the calcium to enter into the cell which explains their tocolytic mechanism [1] (Figure 4). CCBs also inhibit the activating effect of some substances such as *α*_1_-adrenergic receptors, angiotensin II, and endothelin-1 that usually lead to the contraction of smooth muscle fibers [6]. These molecules are arterial vasodilators that decrease the afterload and lead to an increased cardiac flow [2, 10].

Adverse effects linked to *β*_2_-adrenergic agonists, especially APE during pregnancy, resulted in the use of CCBs as tocolytic treatment [11, 12]. In obstetrics, the most studied molecule is nifedipine. Its tocolytic efficacy has been widely evidenced by several randomized trials [13–20]. Several meta-analyses confirm that it is equally efficient as *β*_2_-adrenergic agonists and better tolerated with a decrease in neonatal morbidity [10, 21–24]. These results have led to recommend the use of CCBs as tocolytics in the CNGOF (*Collège National des Gynécologues et Obstétriciens Français*) Clinical Practice Guidelines in 2002 [25]. Clinical studies on nicardipine are rare in the literature. But paradoxically, even if there is no scientific proof of its superiority, nicardipine is more used than nifedipine in French centers because of its IV administration [8]. A first open randomized prospective study by Jannet et al., conducted on 90 patients, compared nicardipine with IV salbutamol; it did not find any difference of tocolytic efficacy but it did find less adverse effects in nicardipine group [26]. Recently, a prospective randomized study conducted on 48 patients with the same molecules confirmed the equal efficacy with a statistically significant difference regarding the adverse effects in favor of nicardipine (8% for nicardipine versus 47% for salbutamol, *P* = 0.02) [27].

CCBs thus appear as preferred tocolytics with a better tolerance than the *β*_2_-adrenergic agonists even if they do not have a marketing authorization [28]. Since they have been used for this indication, minor adverse effects have progressively been described: tachycardia, low blood pressure, palpitations, flushes, headaches, constipation, nausea, and dizziness linked with the vasodilator effect [2].

APE during pregnancy occurs in 0.08–0.5% of the cases regardless of the etiology [29]. Out of 900 patients with a tocolytic indication, nicardipine was used in 742 patients between January 2009 and December 2013 in our hospital (55 births a week). We report here 4 cases of APE in patients who needed to be transferred in ICU regarding the severity of their condition. The incidence of this complication remains low which confirms already published data [30]. In our series, only one patient gave birth to twins whereas most cases in the literature report twin pregnancies [30–33]. No patient had cardiovascular history. The mean time of APE occurrence is 63 hours after nicardipine therapy initiation (Table 1). This mean time is comparable with the published case reports [30–34]. The initial symptoms are not very specific, dyspnea and oxygen desaturation for all cases. Dry cough, signs of acute respiratory distress, thoracic oppression, and palpitations are also found. 50% of our patients had tachycardia. Pulmonary examination disclosed crepitant rales in 50% of cases (Cases 1 and 3). Characteristic crepitant rales can be absent at the beginning but the diagnostic must not be ruled out as they can appear later.

For all 4 patients, the first diagnosis mentioned was pulmonary embolism. One patient even received a heparin bolus with a prescription of heparin at efficient continuous IV doses before she underwent a thoracic angioscan. The normal ECG and troponin rate, when it is performed (50% of cases), rapidly rule out an ischemic origin (Table 1). To our knowledge, only one case of myocardial infarction during tocolytic treatment with nifedipine has been reported in the literature [35]. Laboratory tests revealed an increased BNP each time it was measured (3/4) but a normal result should not rule out the diagnosis as a false negative is possible in case of “flash” APE [36]. Despite the guidelines for pregnant women of the European Society of Cardiology, D-dimer assay which is usually very frequent was not performed in our patients [37]. If a negative result had been obtained, it could have prevented a useless thoracic angioscan. Chest X-ray contributed to APE diagnosis in each case. Regarding echocardiography data, all patients have a maintained LV systolic function. 50% of cases have increased LV filling pressures, and pulmonary hypertension is noted in 75% of cases (Table 1). Tocolytic treatment modalities are reported in Table 2.

The patients receive high doses of nicardipine. However, water and sodium intake linked to nicardipine remains low: a 0.5 mg/mL dilution is used. The maximal volume received equals 1,150 mL over 4 days for the patient who received the highest cumulative dose of nicardipine. In Vaast and Janower series, physiologic saline solution intake due to nicardipine administration reached 1500 mL per day during 4 days [31, 34]. It is always difficult to calculate a precise water balance outside the ICU so we cannot confirm that none of our patients presented blood volume overload out of the nicardipine intake. Our patients presented associated risk factors; a half received tocolytics associated with salbutamol and had a concomitant obstetrical disease; all received a fetal lung maturation corticotherapy which favors sodium and water retention (Table 2). Other published case reports on nicardipine-induced APE are reported in Table 3. APE occurring during a tocolytic treatment with nifedipine or atosiban seem rather rare [38–40].

The pathophysiology of APE during tocolytic treatment with nicardipine is still unclear. The mechanism of action of CCBs does not interact with the determining factors usually at the origin of APE. Maternal physiological adaptation during pregnancy makes the women prone to sodium and water retention. Pregnancy also induces an increase of cardiac flow due to heart rate and systolic ejection volume increase [41]. CCBs from the dihydropyridine family have cardiovascular effects that maximize these physiological adaptations. Their antihypertension action induces a reflex sympathetic stimulation which causes an increase of tachycardia in pregnant women [42, 43]. Nicardipine-induced APE could be an APE due to altered diastolic compliance [44, 45]. Moreover, the sympathetic stimulation activates the renin-angiotensin-aldosterone system which increases sodium and water retention. Finally, in obstetrics, other risk factors are clearly identified: multiple pregnancy, spontaneous premature labor, tocolytic association, fetal lung maturation corticotherapy, preeclampsia or HELLP syndrome, overhydration with physiologic saline solution, valvular heart diseases, and finally infectious environment especially chorioamnionitis [2, 30].

Treatment of nicardipine-induced APE includes discontinuation of the involved medication and a symptomatic treatment associating oxygen therapy with diuretics. With an adapted treatment, the condition improved quickly in our 4 cases; this is comparable to the other cases described in the literature. Some authors suggested using noninvasive ventilation in case of severe hypoxemia in order to postpone orotracheal intubation [46, 47].

## 4. Conclusion

Nicardipine used as tocolytic is more and more frequent in France even if its prescription does not have a marketing authorization. Our series agrees with the 17 cases that have been published since 2004. However pathophysiological mechanisms causing nicardipine-induced APE are still unclear. In the literature, the main reported risk factors for nicardipine-induced APE are spontaneous premature labor, multiple pregnancy, cardiovascular history, concomitant obstetrical disease, in association with other tocolytics, and fetal lung maturation corticotherapy. This complication remains rare but it is advised to use with caution IV nicardipine as a tocolytic in patients with APE risk factors and to avoid association with *β*_2_-adrenergic agonists. A better knowledge of this serious adverse event should allow for an improvement of the diagnostic and therapeutic management in order to avoid useless or even dangerous examination or treatments. Other tocolytics can be used instead such as nifedipine or oxytocin antagonists [48].

## Figures and Tables

**Table 1:** Clinical and paraclinical characteristics in our series.

	Age	Term	Pregnancy	ECG	BNP (ng/L)	Troponin	TTE LVEG	TTE LVFP	TTE PH	N-APE time
Case 1	31	33 WA + 1 d	G1P0	N	2474	ND	70%	↑	Yes	60 h
Case 2	28	26 WA + 1 d	G3P1, TwP	N	3686	ND	>60%	↑	Yes	24 h
Case 3	28	29 WA + 5 d	G2P0	N	660	N	N	N	Yes	≈96 h
Case 4	35	34 WA	G2P0, IVF	N	ND	N	70%	N	No	≈72 h

WA: weeks of amenorrhea; ECG: electrocardiography; TTE: thoracic echocardiography; LVEG: LV ejection fraction; LVFP: LV filling pressures; PH: pulmonary hypertension; N-APE time: time between treatment initiation and acute pulmonary edema occurrence; TwP: twin pregnancy; IVF: *in vitro* fertilization; N: normal; ND: not done.

**Table 2:** Tocolytic treatment modalities and APE risk factors.

	Max flow N (mg/h)	Total dose (mg)	Salbutamol associated	CorticoT	Tachycardia	Other RFs associated
Case 1	3.5	162	No	Yes	No	Preeclampsia
Case 2	4	96	Yes	Yes	Yes	Chorioamnionitis, TwP
Case 3	6	576	No	Yes	Yes	No
Case 4	4	288	Yes	Yes	No	Smoking

N: nicardipine; CorticoT: corticotherapy; RF: risk factors; TwP: twin pregnancy.

**Table 3:** Published case reports on APE occurring during a tocolytic treatment with nicardipine.

Authors	*N*	Premature labor term	Nicardipine maximal flow rate	Associated treatments	Patients characteristics
Vaast et al. [31]	5 cases	29.2 WA (mean)	6 mg/h	IM corticosteroids	2 TwP, 1 TrP, GDM, and cardiovascular history
Bal et al. [49]	1 case	27 WA	2 mg/h	IM corticosteroids	No history
Chapuis et al. [32]	1 case	30 WA + 2 days	2.2 mg/h	IM corticosteroids and IV salbutamol	TwP and 2 abortions
Janower et al. [34]	3 cases	29 WA (mean)	4 mg/h	IM corticosteroids and IV salbutamol	GDM
Philippe et al. [33]	3 cases	31 WA (mean)	MD	IM corticosteroids and IV atosiban	Smoking and 1 TwP
Akerman et al. [30]	4 cases	29 WA (mean)	MD	IM corticosteroids, sup. salbutamol, and IV atosiban	3 TwP and IDD

*N*: number of cases; WA: weeks of amenorrhea; IM: intramuscular; IV: intravenous; TwP: twin pregnancy; TrP: triplet pregnancy; MD: missing data; GDM: gestational diabetes mellitus; sup.: suppository; IDD: insulin dependent diabetes.
